# Effects of cadmium exposure on intestinal microflora of *Cipangopaludina cathayensis*

**DOI:** 10.3389/fmicb.2022.984757

**Published:** 2022-08-08

**Authors:** Jiao-yun Jiang, Wen-hong Li, Yang-yang Wu, Chun-xing Cheng, Quan-qing Ye, Jia-xun Feng, Zhi-xun Xie

**Affiliations:** ^1^College of Life Science and Technology, Guangxi University, Nanning, China; ^2^Guangxi Key Laboratory of Veterinary Biotechnology, Guangxi Veterinary Research Institute, Nanning, China; ^3^Key Laboratory of Ecology of Rare and Endangered Species and Environmental Protection, Ministry of Education, Guangxi Normal University, Guilin, China; ^4^College of Animal Science and Technology, Guangxi University, Nanning, China

**Keywords:** cadmium, *Cipangopaludina cathayensis*, intestinal microbiota, high-throughput sequencing, microbial diversity

## Abstract

As one of the most environmentally toxic heavy metals, cadmium (Cd) has attracted the attention of researchers globally. In particular, Guangxi, a province in southwestern China, has been subjected to severe Cd pollution due to geogenic processes and anthropogenic activities. Cd can be accumulated in aquatic animals and transferred to the human body through the food chain, with potential health risks. The aim of the present study was to explore the effects of waterborne Cd exposure (0.5 mg/L and 1.5 mg/L) on the intestinal microbiota of mudsnail, *Cipangopaludina cathayensis*, which is favored by farmers and consumers in Guangxi. Gut bacterial community composition was investigated using high-throughput sequencing of the V3–V4 segment of the bacterial 16S rRNA gene. Our results indicated that *C. cathayensis* could tolerate low Cd (0.5 mg/L) stress, while Cd exposure at high doses (1.5 mg/L) exerted considerable effects on microbiota composition. At the phylum level, Proteobacteria, Bacteroidetes, and Firmicutes were the dominant phyla in the mudsnail gut microbiota. The relative abundances of Bacteroidetes increased significantly under high Cd exposure (H14) (*p* < 0.01), with no significant change in the low Cd exposure (L14) treatment. The dominant genera with significant differences in relative abundance were *Pseudomonas, Cloacibacterium, Acinetobacter, Dechloromonas*, and *Rhodobacter*. In addition, Cd exposure could significantly alter the pathways associated with metabolism, cellular processes, environmental information processing, genetic information processing, human diseases, and organismal systems. Notably, compared to the L14 treatment, some disease-related pathways were enriched, while some xenobiotic and organic compound biodegradation and metabolism pathways were significantly inhibited in the H14 group. Overall, Cd exposure profoundly influenced community structure and function of gut microbiota, which may in turn influence *C. cathayensis* gut homeostasis and health.

## Introduction

In the wake of rapid industrialization, aquatic ecosystem pollution is becoming severe (Ali et al., 2022). As ubiquitous hazardous pollutants, heavy metals have attracted the attention or researchers globally due to their environmental toxicity. Cadmium (Cd), a non-essential element, usually exists as Cd (II). As one of the most toxic heavy metals, Cd is released into the environment mainly through anthropogenic activities, including electroplating, battery manufacturing, soldering, mining, and agriculture (Burger, 2008). Cd has numerous negative impacts on aquatic animals, including triggering histopathological changes, inducing oxidative stress, causing metabolic disorders, and altering gut microbial community structure (Chang et al., 2019; Liu et al., 2019; Cheaib et al., 2020; Wang et al., 2020a,b). Moreover, Cd is not easily degradable, and can be accumulated in aquatic animals followed by in the human body through the food chain, with potential human health risks (Wang et al., 2022).

Heavy metal pollution is a major environmental issue in China, and heavy metal pollution in aquatic environment is increasing in severity (Chen et al., 2022; Wang et al., 2022). Cd has been identified as one of the major soil contaminants in China (Ministry of Ecology and Environment of the People’s Republic of China, 2014). In particular, Cd contamination in Guangxi province is significantly higher than in other regions in China due to high background geochemical concentrations in the region (Zhao et al., 2015; Wen et al., 2020). In addition, Guangxi province is a key non-ferrous metal production area in China, so that Cd pollution is a major challenge in the province. In early January 2012, the Longjiang River of Guangxi was exposed to serious Cd contamination following an accident, with long-term impacts on the regional aquatic ecosystems (Zhao et al., 2018; Cui et al., 2022). Cd is also the primary heavy metal pollutant in Chinese agricultural land, including paddy soils (Song et al., 2019; Yang et al., 2021). Indeed, people inhabiting such areas with high levels of Cd pollution may be exposed to Cd toxicity, with potential threats to human health (Xu et al., 2018).

The mudsnail, *Cipangopaludina cathayensis* (phylum Mollusca, Gastropoda, Prosobranchia, Mesogastropoda, Viviparidae, and *Cipangopaludina*), is a widely distributed species that can be found in Chinese rivers, lakes, ponds, and other water bodies (Lu et al., 2014). *C. cathayensis* has high protein and low fat content, is rich in umami amino acid, and has high nutritional value, so that it is highly favored among consumers and farmers in China (Luo et al., 2021). Moreover, *C. cathayensis* flesh has been reported to have diverse biological and physiological properties that are beneficial in human disease prevention and treatment (Wang et al., 2016; Zhao et al., 2021).

Indeed, *C. cathayensis* is one of the most popular aquatic animals in China. Particularly in Guangxi province, the snail family Viviparidae is a source of key components of a famous snack, “snail rice noodle,” which represents one of the intangible cultural heritages in China (Luo et al., 2021). In recent years, with the continued increase in “snail rice noodle” consumption, demand for mudsnail and its production has been increasing. Paddy field culture is one of the major ways of mudsnail production in Guangxi. However, Cd pollution has been identified as a serious problem in Guangxi paddy soils and aquatic environments (Zhao et al., 2018; Song et al., 2019; Yang et al., 2021). In addition, considering mudsnail is a benthic organism that is closely associated with paddy soil, it could be exposed to high Cd concentrations, with major threats to food safety (Wang P. et al., 2019). At present, only a few studies had explored the adverse impacts of Cd exposure on the snail family Viviparidae. In addition, current studies have largely focused on the oxidative stress caused by Cd exposure (Hu and Tang, 2012; Zhou and Luo, 2018), so that further investigations on other adverse effects on snails need to be carried out.

The microbiomes associated with aquatic animals, particularly their gut systems, not only participate in digestion but also influence nutrition, growth, reproduction, the immune system, and host vulnerability to disease (Talwar et al., 2018; Chang et al., 2019; Paul and Small, 2019; Duan et al., 2020; Wang et al., 2020a; Diwan et al., 2021). Cd exposure has been reported to significantly affect the gut microbiota of numerous aquatic organisms (Chang et al., 2019; Wang et al., 2020b; Zhang Y. et al., 2020). However, the effects of Cd exposure on the intestinal microbiota of *C*. *cathayensis* remain unclear. To address the knowledge gap, in the present study, *C*. *cathayensis* individuals were exposed to two doses (0.5 mg/L and 1.5 mg/L) of cadmium chloride (CdCl_2_⋅2.5H_2_O) for > 14 days. The aim of the present study was to investigate the effect of Cd on *C. cathayensis* gut microbiota composition and diversity.

## Materials and methods

### Ethics statement

The experimental protocol for snail acclimation and experimentation was approved by the Animal Ethics committee of Guangxi Normal University, Guilin, Guangxi, China (No. 202207-02).

### Experimental snail and treatment

Adult snails (*C. cathayensis*) were obtained from Juhe Agricultural Development Cooperatives (25.75° N, 109.38° E), Sanjiang District, Liuzhou City, Guangxi, China. They were then transferred to the laboratory, and acclimated to the experimental conditions at a temperature 24.0 ± 1.0°C, under a 12-h/12-h light/dark cycle in a 50-L (65 × 41 × 20 cm) plastic tank for 2 weeks. During the acclimation period, specimens were fed with commercial ground fish food (Tongwei, Chengdu, Sichuan, China) once a day at 0.5% of their body weight. The tank water was changed partially (30%) every day.

After a 2-week acclimation period, 225 snails were divided randomly into three groups and placed in plastic tanks, with three replicates (25 snails per tank) in each treatment. CdCl_2_⋅2.5H_2_O (Silong, Shantou, Guangdong, China) was dissolved in deionized water to prepare stock solution with a final concentration of 900 mg/L. The 0.5 mg/L and 1.5 mg/L Cd doses were selected according to previous studies (Hu and Tang, 2012). The three treatments in the present study included the control treatment (**CK14**: with no Cd supplementation), low Cd concentration exposure treatment (**L14**: 0.5 mg/L), and high Cd concentration exposure treatment (**H14**: 1.5 mg/L). Other experimental conditions were consistent with those in the acclimation phase. During the experimental period, one-third of the water in the tank was replaced every day by adding fresh water or water with a similar concentration. The experiment lasted 2 weeks, as severe mortality occurred at 14 -day in the H14 treatment (Supplementary Table S1).

### Sample collection

Snail intestine samples were collected on day 14 and used to determine gut microbiota composition and diversity. The guts of three snails were pooled as a single sample, to ensure sample adequacy, with three biological replicates in each treatment. Briefly, the samples were wiped with 75% ethanol before the snails were removed from the shell. Subsequently, the snails were dissected and the guts extracted and rinsed with sterile water three times. The gut samples were flash frozen using liquid nitrogen and stored at −80°C for subsequent analyses.

### DNA extraction, bacterial 16S rRNA amplification, and sequencing

Total genomic DNA (gDNA) of the gut microbiota were extracted using a Fast DNA SPIN Extraction Kit (MP Biomedicals, United States) according to the manufacturer’s protocol. The V3–V4 regions of the bacterial 16S rRNA genes were amplified by PCR using universal bacterial primers (338F: 50-ACTCCTACGGGAGGGAGCA-30, 806R: 50-GGACTACHVGGGTWTCTAAT-30). The PCR cycle conditions for each sample were as follows: an initial denaturation at 95°C for 5 min; 25 cycles of denaturation at 95°C for 30 s, annealing at 55°C for 30 s, and extension at 72°C for 30 s, with a final extension at 72°C for 5 min. PCR products were purified and quantified using an AxyPrep DNA Gel Extraction Kit (Axygen, Union City, NJ, United States) and a Quant-iT PicoGreen dsDNA Assay Kit (Invitrogen, Waltham, MA, United States), respectively. A TruSeq Nano DNA LT Library Prep Kit (Illumina, United States) was used to establish the DNA library. The library was sequenced using a MiSeq Reagent Kit v3 (6,000-cycles-PE) (Illumina, United States) on a MiSeq platform by Personal Biotechnology Co., Ltd. (Shanghai, China). The raw reads were deposited into the NCBI Sequence Read Archive database (PRJNA837347).

### Sequence processing

The sequencing data were processed using Quantitative Insights Into Microbial Ecology 2 (QIIME2 v2019.4^1^). Briefly, Cutadapt (version 3.7) was used to filter and trim PCR primers from the raw reeds. DADA2 was used for quality control (Callahan et al., 2016), removing chimera sequences, and determining the sequence variants. Taxonomy was assigned using the DADA2 pipeline, which implements the Naive Bayesian Classifier using the DADA2 default parameters based on the Greengenes database (Release 13.8^2^). Subsequently, the sequences were rarefied using the feature-table rarefy command in QIIME2.

### Data analysis

All sequence analysis steps were performed using QIIME2 and R v3.2.0 (R Foundation for Statistical Computing, Vienna, Austria). The rarefaction curve was generated based on Amplicon Sequence Variants (ASVs) at a 97% similarity cut-off level. For alpha diversity analyses, Chao 1, Observed_species, Shannon, and Simpson indices were calculated using QIIME2 (for calculation methods^3^). Significance between groups was tested using the Kruskal–Wallis *H* test and the Dunn test. Beta diversity was calculated using weighted Bray-Curtis distance matrix and visualized with Principal Coordinates Analysis (PCoA). Hierarchical clustering using Bray–Curtis distances based on the relative abundances of species was performed to cluster the dataset. A Venn diagram was drawn using the “VennDiagram” package in R v3.2.0 (R Statistical Foundation). The functional profiles of microbial communities were predicted using PICRUSt2 (Phylogenetic Investigation of Communities by Reconstruction of Unobserved States^4^). The predicted genes and their respective functions were annotated using the Kyoto Encyclopedia of Genes and Genomes (KEGG) database^5^. Differences between populations were analyzed using one-way Analysis of Variance. Results were considering statistically significant at *p* < 0.05. The values are expressed as mean ± SD (Standard deviation).

## Results

### Relative abundance

**FIGURE 1:**
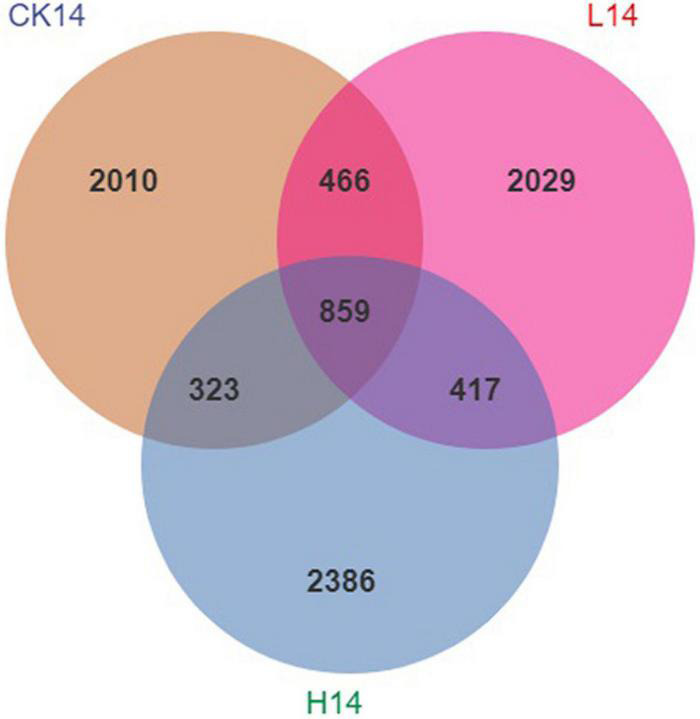
Venn diagram analysis depicting the numbers of shared and unique Amplicon Sequence Variants (ASVs) among the control (CK14), 0.5 mg/L (L14), and H14 (1.5 mg/L) treatments.

After normalization, there were 833,989 sequences across all snail gut contents sampled, with an average of 92,665 sequences per sample (minimum of 55,824 sequences per sample and maximum of 145,373 sequences per sample, see Supplementary Table S2). Rarefaction curves indicated that all samples reached the saturation phase (Supplementary Figure S1). There were 14,951 ASVs derived from all samples; the CK14, L14, and H14 treatments had 4,625, 5,121, and 5,205 ASVs per sample, respectively. Moreover, 859 ASVs were shared among the three treatments, while 2,010, 2,029, and 2,386 ASVs were unique to the CK14, L14, and H14 treatments, respectively (Figure 1).

### Intestinal microflora diversity

**FIGURE 2:**
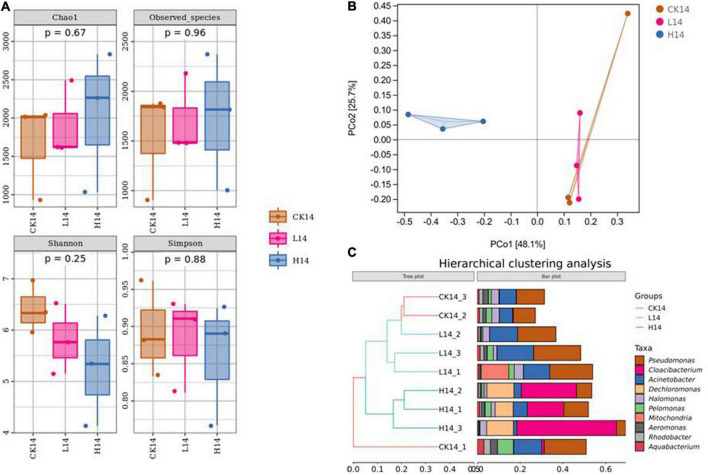
Intestinal microbiome diversity in the control (CK14), 0.5 mg/L (L14) and H14 (1.5 mg/L) groups. **(A)** α-diversity comparisons in the intestinal microflora among the CK14, L14, and H14 groups. **(B)** Bray–Curtis distances were calculated and visualized through Principal Coordinate Analysis (PCoA) (Ellipses were drawn with 95% confidence intervals). **(C)** Hierarchical cluster analysis of the Bray–Curtis distances generated from taxa tables showed Amplicon Sequence Variant (ASV) similarity across microbial communities among different groups.

To compare bacterial community diversity across different groups, alpha-diversity and beta-diversity were evaluated. There were no significant differences in Chao 1 index, Observed_species index, Shannon index, and Simpson index among the three groups (*p* > 0.05) (Figure 2A and Supplementary Table S3). In a beta-diversity analysis (PCoA based on Bray–Curtis), the L14 and CK14 treatments were clustered together and could not be distinguished, whereas the H14 group was distinct from the L14 and CK14 groups, with the following main principal component (PC) scores: PC1 = 48.1%, PC2 = 25.7% (Figure 2B). In addition, according to the hierarchical clustering tree results, ASVs from *C*. *cathayensis* in the high Cd exposure group were clustered in one group based on similarity, while the control and low Cd exposure groups clustered into one independent group, excluding one control sample (Figure 2C). The results indicate that the high Cd exposure treatment had more severe effects on the diversity of the *C. cathayensis* microbiome than the low Cd exposure treatment.

### Gut microbiota community structure

**FIGURE 3:**
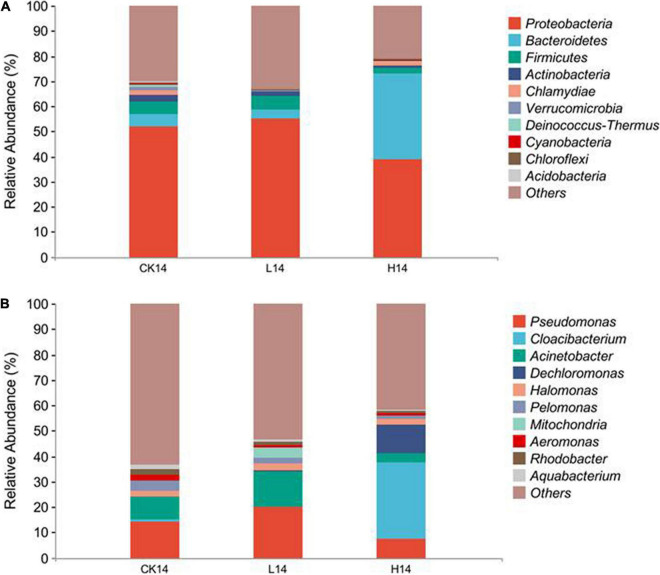
Compositions of the intestinal microflora among the control (CK14), 0.5 mg/L (L14), and H14 (1.5 mg/L) treatments. **(A)** Compositions of the intestinal microflora at the phylum level. **(B)** Compositions of the intestinal microflora at the genus level. The top ten abundant genera (higher than 1% in at least one sample) are shown in the figure and the rest are indicated as “Others.”

In total, 25 phyla, 50 classes, 115 orders, 185 families, 324 genera, and 90 species were identified. At the phylum level, Proteobacteria was the most abundant phylum across all three treatments (51.9% in CK14, 55.2% in L14, and 38.9% in H14), the other two prevalent phyla were Bacteroidetes and Firmicutes (Figure 3A and Supplementary Table S4). In addition, Bacteroidetes abundance in the H14 treatment was significantly higher than that in the C14 treatment (*p* < 0.01), although there was no significant difference between the L14 and control treatments (Figure 3A and Supplementary Figure S2a). At the genus level, *Pseudomonas, Cloacibacterium, Acinetobacter, Dechloromonas, Halomonas, Pelomonas, Mitochondria, Aeromonas, Rhodobacter*, and *Aquabacterium* were the dominant (Figure 3B and Supplementary Table S5). *Pseudomonas* relative abundance was higher in the L14 treatment than in the C14 treatment, although the difference was not significant (Figure 3B and Supplementary Figure S2b). Conversely, *Pseudomonas* relative abundance was lower in the H14 treatment than in the C14 treatment, although the difference was not significant (Figure 3B and Supplementary Figure S2b). However, *Pseudomonas* relative abundance decreased with an increase in Cd concentration (*p* < 0.01) in the H14. *Acinetobacter* exhibited a similar trend (Figure 3B and Supplementary Figure S2c). In addition, *Rhodobacter* relative abundance was significantly lower in the H14 treatment than in the C14 treatment (*p* < 0.05). *Rhodobacter* relative abundance was also lower in the L14 treatment than in the C14 treatment, although the difference was not significant (Figure 3B and Supplementary Figure S2d). On the contrary, *Cloacibacterium* and *Dechloromonas* were significantly enriched in the H14 treatment (*p* < 0.05), although there was no significant indifference between the L14 treatment and the CK14 treatment (Figure 3B and Supplementary Figures S2e,f). The results were consistent with beta diversity analysis results (Figures 2B,C). Cd exposure at low doses had minimal effect on snail gut microbial diversity, whereas high Cd stress influenced snail gut microbial diversity considerably.

### Prediction of microbial community function

**FIGURE 4:**
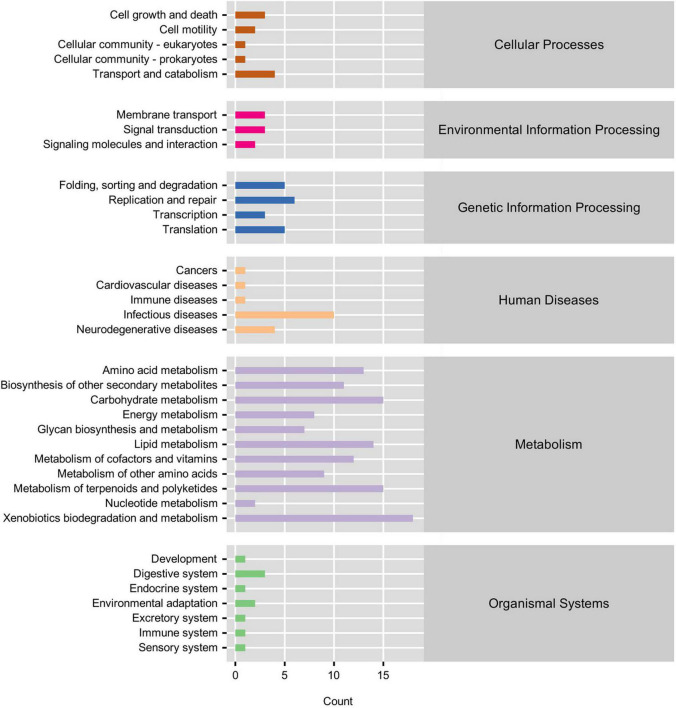
Functional annotations and abundance information about the intestinal microbiota at KEGG level 1 and level 2. The gene function is showed as color-bars (level 1). The detailed pathways are shown on the left side (level 2).

**FIGURE 5:**
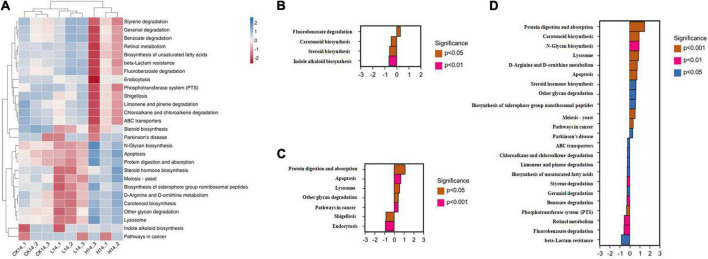
Intestinal microbiota predictive metabolic functions from the Kyoto Encyclopedia of Genes and Genomes (KEGG) database in all samples. **(A)** Heatmap of the significant differential pathways among the control (CK14), 0.5 mg/L (L14), and H14 (1.5 mg/L) treatments. **(B)** Significant different pathways between the control and L14 treatments. **(C)** Significantly different pathways between the control and H14 treatments. **(D)** Significantly different pathways between the L14 and H14 treatments.

PICRUSt functional prediction and KEGG pathway enrichment analysis results showed that the main functional categories included five cellular processes pathways, three environmental information processing pathways, four genetic information processing pathways, five human disease pathways, 11 metabolism pathways, and seven organismal system pathways (Figure 4). In the L14 treatment, the fluorobenzoate degradation pathway was enriched; on the contrary, carotenoid biosynthesis, steroid biosynthesis, and indole alkaloid biosynthesis pathways were significantly down-regulated in the L14 treatment compared with in the control treatment (Figures 5A,B). In the H14 treatment, five pathways (protein digestion and absorption, apoptosis, lysosome, other glycan degradation, and pathways in cancer) were significantly up-regulated, whereas shigellosis and endocytosis pathways were decreased relative to the control group (Figures 5A,C). In addition, notably, compared with in the L14 treatment, some xenobiotic and organic compound biodegradation and metabolism pathways were significantly reduced in the H14 treatment (Figures 5A,D).

## Discussion

### Intestinal microbial diversity

Cadmium is undoubtedly an environmental contaminant. Previous studies have demonstrated that Cd exposure could alter intestinal flora composition in aquatic animals (Chang et al., 2019; Wang et al., 2020b; Zhang Y. et al., 2020). In the present study, intestinal microbiota in *C*. *cathayensis* was investigated using high-throughput 16S rRNA gene sequencing. Our results suggested no significant difference in alpha diversity among the three treatments (Figure 2A). The results are inconsistent with the findings of previous studies that have reported that Cd exposure altered the alpha diversity of gut microbiota (Ya et al., 2019; Zhang Y. et al., 2020), even under relatively low Cd concentration (Chang et al., 2019). Furthermore, the PCoA analysis results showed that gut microbial community structure in the high Cd exposure treatment was distinct from that in the low Cd exposure and control treatments, whereas the taxonomic groups in the latter two treatments were clustered together (Figure 2B). In addition, hierarchical clustering tree construction revealed that the intestinal samples of the three exposure treatments were clustered into two independent groups, excluding one control sample (Figure 2C), implying that Cd exposure at high dose (1.5 mg/L) exerted greater effects on the microbiota composition in *C*. *cathayensis*. The results indicate that *C*. *cathayensis* could potentially tolerate low Cd stress. Gut microbiome systems of aquatic animals participate in various processes, including nutrition, growth, immunity, and disease resistance (Talwar et al., 2018; Chang et al., 2019; Paul and Small, 2019; Duan et al., 2020; Wang et al., 2020a; Diwan et al., 2021). In the present study, intestinal microbiota structure was altered in the *C*. *cathayensis* gut under high Cd exposure when compared with in the control treatment (Figures 2B,C). Consequently, alteration of intestinal microbial community structure following Cd exposure could induce adverse effects on *C*. *cathayensis* health.

### Effects of Cd exposure on gut microbial community

In the present study, phylum Proteobacteria was the dominant phylum across all three groups. The results are consistent with the findings of recent studies in other *Cipangopaludina* species (Zhou K. Q. et al., 2022; Zhou Z. H. et al., 2022). The other two dominant phyla were Bacteroidetes and Firmicutes, which are consistent with the findings of previous studies that have reported that the major bacterial phyla in the gut of aquatic animals, including fish, crustaceans, and mollusks are Proteobacteria, Bacteroidetes, and Firmicutes (Chang et al., 2019; Liu et al., 2019; Wang et al., 2020b; Zhang Y. et al., 2020; Zhou Z. H. et al., 2022). However, the two phyla were not dominant in a closely related species, *Cipangopaludina chinensis* (Zhou K. Q. et al., 2022; Zhou Z. H. et al., 2022). The result implies that although the two species are closely related, they may have different strategies of responding to Cd stress. Nevertheless, further research is required to investigate the factors responsible for the difference between the two species. Compared to that in the control, the abundance of Bacteroidetes was significantly higher in the H14 treatment, although there was no significant difference between the L14 and control treatments. Bacteroidetes, the largest phylum of Gram-negative bacteria in the human gastrointestinal tract microbiome, has the potential to secrete surface lipopolysaccharides and toxic proteolytic peptides, which can cause inflammation in the gut (Lukiw, 2016). The elevated phylum Bacteroidetes abundance in H14 treatment indicated that high Cd exposure has potential adverse effects on *C*. *cathayensis* health.

Genus *Pseudomonas*, which has been identified as bacterial pathogen in teleosts, exists widely in aquatic environments and in the gut of aquatic animals (Llewellyn et al., 2014; Xu et al., 2015; Dehler et al., 2017; Diwan et al., 2021). *Pseudomonas* has also been observed to increase in the guts of different vertebrates, including fish and amphibians, following Cd exposure (Chang et al., 2019; Ya et al., 2019; Cheaib et al., 2020). Furthermore, *Pseudomonas* outbreaks have been reported in aquacultured animals (Llewellyn et al., 2014; Xu et al., 2015). However, *Pseudomonas* are also considered probiotics for application in aquaculture (Wang A. R. et al., 2019), that can chelate or oxidize heavy metals, thereby facilitating heavy metal excretion and minimizing the exposure of organisms to heavy metals (Duan et al., 2020; Arun et al., 2021). In the present study, *Pseudomonas* relative abundance in the L14 treatment was higher than that in the control treatment, although the difference was not significant. However, *Pseudomonas* relative abundance in the H14 treatment was lower than that in the control treatment, although not significant. Notably, *Pseudomonas* abundance decreased with an increase in Cd concentration, suggesting that *Pseudomonas* could play a role in Cd toxicity removal. However, probiotics contents decreased with an increase in Cd concentration, which could adversely affect Cd toxicity tolerance in mudsnail. Indeed, high snail mortality was observed in the H14 treatment but not in the L14 treatment. The results further confirm our postulation above that *C*. *cathayensis* could acclimate to low Cd concentration, potentially by accumulating *Pseudomonas*. *Acinetobacter* are putative pathogens. Their abundance increased significantly in the gut of Nile tilapia (Zhai et al., 2016) and common carp (Chang et al., 2019) following Cd exposure, and greatly increased in methyl-mercury (MeHg)-exposed fish (Bridges et al., 2018). Studies have shown that *Acinetobacter* may exert adverse effects on fish health (Wu et al., 2013; Wang et al., 2020c). Consistent with the previous findings, *Acinetobacter* increased in the L14 treatment, although not significantly. However, when Cd concentration reached 1.5 mg/L, *Acinetobacter* reduced considerably, which may be related to the extremely high Cd content (Wang et al., 2020c). Similarly, *Rhodobacter* significantly decreased in the H14 treatment. Decreased *Rhodobacter* abundance has been reported to reduce growth (Liu H. S. et al., 2021) and to have adverse effects on fish innate immunity (Wang et al., 2020c), resulting in increased vulnerability to disease (She et al., 2017; Liu F. P. et al., 2021). Indeed, *Rhodobacter* is a candidate probiotic for fish (Ye et al., 2019). However, some studies have found that higher abundances of such bacteria could be associated with diseased intestines (Tran et al., 2018), and they could cause neurotoxicity in the hosts (Bridges et al., 2018; Arun et al., 2021). Such findings illustrate the importance of intestinal bacterial community homeostasis in hosts. Decreased *Rhodobacter* abundance in the present study suggest that intestinal function could have been impaired in *C*. *cathayensis* exposed to Cd, which could result in disease outbreaks under natural conditions (She et al., 2017; Liu F. P. et al., 2021).

In the present study, *Cloacibacterium*, a key genus in the phylum Bacteroidetes implicated in xenobiotic metabolism and metal removal (Nouha et al., 2016; Duan et al., 2020), increased significantly in the H14 treatment. *Cloacibacterium* has been used to detoxify MeHg in MeHg-exposed fish (Bridges et al., 2018). Enrichment of *Cloacibacterium* has been reported to be an important feature under MeHg-induced neurotoxicity (Bridges et al., 2018). In addition, in the present study, *Dechloromonas* abundance increased in the H14 treatment. *Dechloromonas*, which belongs to the phylum Proteobacteria and is considered a Cd-resistant microorganism (Zhang et al., 2019), could efficiently degrade polycyclic aromatic hydrocarbons during sludge composting (Lu et al., 2019; Che et al., 2021), and participate in organic matter degradation in aquaculture pond sediment (Zhang K. K. et al., 2020). *Cloacibacterium* and *Dechloromonas* enrichment in *C*. *cathayensis* gut in the present study highlight their potential roles in Cd detoxification, which merit further study.

### Intestinal microbiome function

Our function prediction analysis of the gut microbiota showed that most of the genes encoded by the *C*. *cathayensis* gut microbiota were related to metabolism, followed by organismal systems, cellular processes pathways, human diseases pathways, genetic information processing pathways, and environmental information processing pathways (Figure 4). The intestines are essential organs involved in the metabolism of nutrients (Liu et al., 2022). The results suggest that Cd exposure may alter gut microbial function and host metabolism. In addition, function prediction results showed that, compared with the control treatment, only one pathway related to fluorobenzoate degradation was enriched in the L14 treatment, whereas more pathways were enriched in the H14 treatment (Figure 5), including protein digestion and absorption, apoptosis, lysosome, other glycan degradation, and pathways in cancer. Fluorobenzoate is the sole carbon and energy source for *Pseudomonas* (Kalpit et al., 1988). The fluorobenzoate degradation pathway was enriched in the L14 treatment, which is consistent with the increasing trends in *Pseudomonas* abundance observed in the treatment group. The cell apoptosis pathway is usually activated following disease infection (Qiu et al., 2020) or exposure to adverse environmental factors (Chen et al., 2021). Furthermore, lysosomes not only play a central role in cell decomposition but also participate in metabolism, membrane repair, and cell death (Serrano-Puebla and Boya, 2015), and lysosome metabolic pathways are closely related to cell apoptosis (Guo et al., 2017). In the present study, lysosome pathway and cell apoptosis pathway were both enriched in the H14 treatment, which suggests that high Cd exposure may exert more adverse effects on gut microbes of snails than low Cd exposure. Furthermore, pathways in cancer were also enriched in the H14 treatment. The results above partially explain our hypothesis above that *C*. *cathayensis* has a capacity to acclimate to low Cd stress.

It is also worth noting that pathways associated with xenobiotic and organic compound biodegradation and metabolism, including chloroalkane and chloroalkene degradation, benzoate degradation, fluorobenzoate degradation, styrene degradation, limonene and pinene degradation, geraniol degradation, were significantly inhibited in the H14 treatment in the present study, when compared to in the L14 treatment. Chloroalkane, chloroalkene, benzoate, fluorobenzoate and styrene are xenobiotics found in the environment (Vera et al., 2022). However, the pathways associated with the degradation of the xenobiotics were down-regulated in the H14 treatment, which suggested that the capacity of elimination of the compounds decreased following exposure to high Cd doses (Gu et al., 2017; Vera et al., 2022). Limonene and pinene are considered anti-inflammatory molecules; the down-regulation of the limonene and pinene degradation pathway in the H14 treatment could have increased the levels of limonene and pinene, which could have antagonized the inflammatory response caused by Cd stress (Han et al., 2021). Geraniol is another carbon and energy source for some *Pseudomonas* species (Vandenbergh and Wright, 1983; Zhu et al., 2020). The decline in the geraniol degradation pathway in the present study could be attributed to the decreased contents of the genus *Pseudomonas*. Overall, according to the results of the present study, Cd exposure disrupts gut microbial community structure and their potential functions, and could in turn, adversely influence *C*. *cathayensis* health.

## Conclusion

Our results revealed that Cd exposure could significantly alter the structure and function of intestinal microbial communities, which may in turn influence *C. cathayensis* gut homeostasis and health. To the best of our knowledge, this is the first study to explore the effects of Cd exposure on the intestinal microbiota of *C. cathayensis*. The results obtained in this study provide insights into the mechanisms associated with the response of the intestinal microbiota of *C. cathayensis* to Cd pollution. However, obtaining the 16S rRNA gene sequences through the Illumina HiSeq platform has limitations. In the present study, we did not isolate and identify the putatively pathogenic and putatively beneficial bacteria, which warrants further research.

## Data availability statement

The datasets presented in this study can be found in online repositories. The names of the repository/repositories and accession number(s) can be found below: https://www.ncbi.nlm.nih.gov/, PRJNA837347.

## Ethics statement

The animal study was reviewed and approved by Animal Ethics Committee of Guangxi Normal University.

## Author contributions

Z-XX, J-XF, and J-YJ conceived and designed the study. Z-XX, J-YJ, and W-HL contributed reagents and materials. J-YJ and Y-YW analyzed the data. Y-YW and C-XC performed the gut extraction. Y-YW, C-XC, and Q-QY cultured the snails. J-YJ and W-HL wrote the manuscript. All authors read and approved the final manuscript.

## Footnotes

1
http://qiime.org

2
http://greengenes.secondgenome.com/

3
http://scikit-bio.org/docs/latest/generated/skbio.diversity.alpha.html#module-skbio.diversity.alpha

4
https://github.com/picrust/picrust2

5
http://www.genome.jp/kegg/pathway.html

## Supplementary material

Supplementary Figure S1 Rarefaction curves for all the analyzed samples. Different samples are represented by curves of different colors.

Supplementary Figure S2 Relative abundance of intestinal bacteria. **(a)** The relative abundance of phylum Bacteroidetes; **(b)** genus *Pseudomonas*; **(c)** genus *Acinetobacter*; **(d)** genus *Rhodobacter*; **(e)** genus *Cloacibacterium*; **(f)** and genus *Dechloromonas*. Asterisks (*) indicate *p*-values < 0.05.
