# Supplementary figures and images for: Effects of cadmium exposure on intestinal microflora of Cipangopaludina cathayensis
Source: Front Microbiol. 2022 Aug 8;13:984757. doi: 10.3389/fmicb.2022.984757 (PMC9393624; doi:10.3389/fmicb.2022.984757)

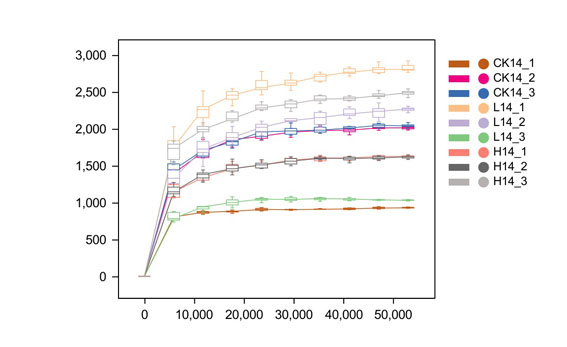

Supplement: Supplementary Figure S1 — Rarefaction curves for all the analyzed samples. Different samples are represented by curves of different colors. [file Image_1.JPEG]

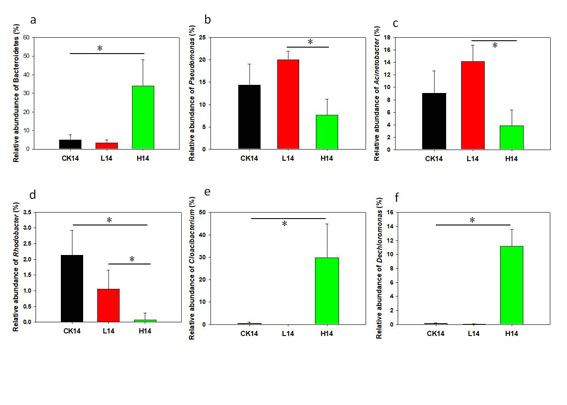

Supplement: Supplementary Figure S2 — Relative abundance of intestinal bacteria. (a) The relative abundance of phylum Bacteroidetes; (b) genus Pseudomonas; (c) genus Acinetobacter; (d) genus Rhodobacter; (e) genus Cloacibacterium; (f) and genus Dechloromonas. Asterisks (*) indicate p-values < 0.05. [file Image_2.JPEG]
