# Supplementary material for: Effects of cadmium exposure on intestinal microflora of Cipangopaludina cathayensis
Source: Front Microbiol. 2022 Aug 8;13:984757. doi: 10.3389/fmicb.2022.984757 (PMC9393624; doi:10.3389/fmicb.2022.984757)
Supplement: Supplementary file 3 [file Data_Sheet_1.docx]

**Supplemental Information**

**Table S1. Death rates (%) of *Cipangopaludina cathayensis* under cadmium stress.**

| Group | Death rates (%) | | |
| --- | --- | --- | --- |
|  | day 3 | day 7 | day 14 |
| Control | 0 | 0 | 0 |
| L14 | 0 | 0 | 6.8±2.3 |
| H14 | 0 | 27.8±6.3 | 54.3±16.9 |

**Table S2. Sequence numbers derived from nine samples.**

| Sample ID | Input | Filtered | Denoised | Merged | Non-chimeric | Non-singleton |
| --- | --- | --- | --- | --- | --- | --- |
| CK14_1 | 96753 | 89815 | 89298 | 87024 | 85713 | 85655 |
| CK14_2 | 87984 | 76515 | 73328 | 67467 | 62806 | 62417 |
| CK14_3 | 96376 | 84781 | 81704 | 75281 | 69420 | 69057 |
| L14_1 | 94692 | 82141 | 78818 | 73451 | 65477 | 65081 |
| L14_2 | 96105 | 81740 | 78817 | 74361 | 68970 | 68657 |
| L14_3 | 176618 | 156845 | 152559 | 145419 | 140801 | 140446 |
| H14_1 | 187149 | 169193 | 164447 | 156380 | 146038 | 145373 |
| H14_2 | 182083 | 167077 | 162874 | 155824 | 142099 | 141479 |
| H14_3 | 79397 | 67557 | 65701 | 62208 | 56091 | 55824 |

**Table S3. The alpha-diversity indexes of different samples under a 97% similarity cut-off.**

| Sample | Chao1 | Observed_species | Shannon | Simpson |
| --- | --- | --- | --- | --- |
| CK14_1 | 932.762 | 905.6 | 6.96714 | 0.961657 |
| CK14_2 | 2014.7 | 1841.3 | 5.95325 | 0.832953 |
| CK14_3 | 2040.73 | 1876.6 | 6.33026 | 0.882758 |
| L14_1 | 1624.36 | 1479.2 | 5.76501 | 0.910591 |
| L14_2 | 1615.74 | 1482.5 | 5.15267 | 0.811341 |
| L14_3 | 2493.8 | 2181.5 | 6.5085 | 0.929922 |
| H14_1 | 2831.43 | 2373.4 | 6.27748 | 0.924488 |
| H14_2 | 2265.61 | 1816.6 | 5.34004 | 0.890473 |
| H14_3 | 1032.85 | 1003.1 | 4.13539 | 0.767315 |

**Table S4. Relative abundance of intestinal bacteria at phylum level.**

| ID | CK14_1 | CK14_2 | CK14_3 | L14_1 | L14_2 | L14_3 | H14_1 | H14_2 | H14_3 |
| --- | --- | --- | --- | --- | --- | --- | --- | --- | --- |
| Proteobacteria | 0.75149145 | 0.38976561 | 0.41416511 | 0.61561746 | 0.4316239 | 0.6073082 | 0.4760788 | 0.3904184 | 0.3000322 |
| Bacteroidetes | 0.08034557 | 0.02842174 | 0.03935879 | 0.02636714 | 0.022911 | 0.0517138 | 0.2196144 | 0.3044127 | 0.4940169 |
| Firmicutes | 0.07328235 | 0.03074483 | 0.0420667 | 0.05431693 | 0.0404474 | 0.0767982 | 0.039464 | 0.0195011 | 0.0125573 |
| Actinobacteria | 0.03612165 | 0.02638704 | 0.02681843 | 0.01906855 | 0.0130795 | 0.0156003 | 0.0144662 | 0.0084041 | 0.0090642 |
| Chlamydiae | 0.00186796 | 0.00073698 | 0.04859754 | 0.00192068 | 0.000335 | 0.0023568 | 0.0092658 | 0.028089 | 0.00249 |
| Verrucomicrobia | 0.00957329 | 0.01417883 | 0.01479937 | 0.00093729 | 0.0006117 | 0.0014881 | 0.0024076 | 0.0020286 | 0.0004299 |
| Deinococcus-Thermus | 0.01548071 | 0.00821891 | 0.00754449 | 0.00119851 | 0.0018352 | 0.0029335 | 0.0021668 | 0.0014066 | 0.0009673 |
| Cyanobacteria | 0.00451813 | 0.00121762 | 0.00205627 | 0.00118314 | 0.0010924 | 0.0029549 | 0.0014239 | 0.0135427 | 0.0003045 |
| Chloroflexi | 0.00422626 | 0.00306006 | 0.00325818 | 0.00262749 | 0.0012963 | 0.0032041 | 0.004258 | 0.0025728 | 0.0012719 |
| Acidobacteria | 0.00728504 | 0.00288383 | 0.00185354 | 0.00036877 | 0.0004078 | 0.0012389 | 0.001362 | 0.0008553 | 0.0001971 |
| Others | 0.0158076 | 0.49438454 | 0.39948159 | 0.27639403 | 0.4863597 | 0.2344033 | 0.2294924 | 0.2287689 | 0.1786687 |

**Table S5. Relative abundance of intestinal bacteria at the genus level.**

| ID | CK14_1 | CK14_2 | CK14_3 | L14_1 | L14_2 | L14_3 | H14_1 | H14_2 | H14_3 |
| --- | --- | --- | --- | --- | --- | --- | --- | --- | --- |
| Pseudomonas | 0.1956804 | 0.1040902 | 0.1314856 | 0.2004886 | 0.179734 | 0.2194794 | 0.1146568 | 0.0714382 | 0.0429744 |
| Cloacibacterium | 0.0120717 | 0.0025153 | 0.0011295 | 9.22E-05 | 0.000102 | 0.0001922 | 0.1710703 | 0.2586038 | 0.4650688 |
| Acinetobacter | 0.1308155 | 0.0621626 | 0.0785004 | 0.123062 | 0.1306495 | 0.1713185 | 0.0657412 | 0.0334679 | 0.0162654 |
| Dechloromonas | 0.0016812 | 0.0005768 | 0.0020273 | 0.0005224 | 0.0002913 | 0.0009256 | 0.0842522 | 0.1267467 | 0.1249104 |
| Halomonas | 0 | 0.0347662 | 0.0353476 | 0.0423011 | 0.0311403 | 0.0164191 | 0.0186486 | 0.0125178 | 0.031456 |
| Pelomonas | 0.074193 | 0.026387 | 0.0133803 | 0.0254452 | 0.0064087 | 0.0282244 | 0.027997 | 0.0117261 | 0.0017913 |
| Mitochondria | 0.0004203 | 0.0004646 | 0.0003765 | 0.1289777 | 0.001005 | 0.0011606 | 0.001534 | 0.0003463 | 0.0001254 |
| Aeromonas | 0.0327243 | 0.0152523 | 0.0255876 | 0.0039182 | 0.0056076 | 0.0150165 | 0.0153055 | 0.0066794 | 0.0041022 |
| Rhodobacter | 0.0303193 | 0.0151561 | 0.0183182 | 0.0055469 | 0.0086663 | 0.0171525 | 0.0084748 | 0.0085879 | 0.0046038 |
| Aquabacterium | 0.0312766 | 0.0107663 | 0.0082541 | 0.0090349 | 0.0044569 | 0.0135426 | 0.0116322 | 0.0050608 | 0.0020063 |
| Others | 0.4908178 | 0.7278626 | 0.6855931 | 0.4606106 | 0.6319385 | 0.5165687 | 0.4806876 | 0.4648252 | 0.306696 |
